# (2,2′-Bipyridine-κ^2^ *N*,*N*′)bis­(3-meth­oxy­benzoato-κ^2^ *O* ^1^,*O* ^1′^)copper(II) mono­hydrate

**DOI:** 10.1107/S1600536811005563

**Published:** 2011-02-19

**Authors:** Ming-Hao Lin, Jing-Fan Zhou, Bin-Bin Liu, Jian-Li Lin

**Affiliations:** a State Key Laboratory Base of Novel Functional Materials and Preparation Science, Center of Applied Solid State Chemistry Research, Ningbo University, Ningbo, Zhejiang, 315211, People’s Republic of China

## Abstract

The title compound, [Cu(C_8_H_7_O_3_)_2_(C_10_H_8_N_2_)]·H_2_O, is comprised of a Cu^II^ ion, two 3-meth­oxy­benzoate ligands, a 2,2′-bipyridine (bipy) ligand and one uncoordinated water mol­ecule. The Cu^II^ ion and the water O atom lie on a twofold axis. The Cu^II^ ion exhibits a six-coordinate distorted octa­hedral geometry, with two N atoms from the bipy ligand [Cu—N = 1.9996 (16) Å] and four O atoms from two 3-meth­oxy­benzoate ligands [Cu—O = 1.9551 (15) and 2.6016 (16) Å]. The mol­ecules are linked by O—H⋯O and C—H⋯O hydrogen bonds, forming a three-dimensional network.

## Related literature

For hydrogen bonds and crystal engineering, see: Aakeröy & Seddon (1993 ▶). For potential applications of transition metal complexes, see: Liu *et al.* (2007 ▶); Shibasaki & Yoshikawa (2002 ▶). For carboxylate compounds with six-coordinate metal atoms, see: Liu *et al.* (2010 ▶); Su *et al.* (2005 ▶).
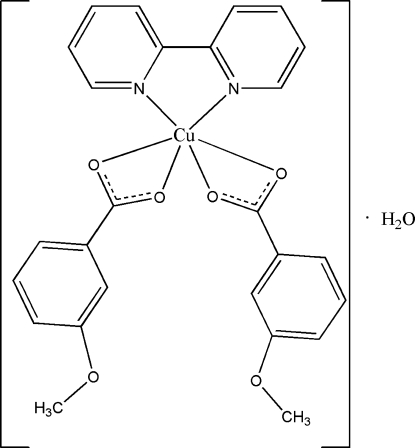


## Experimental

### 

#### Crystal data

[Cu(C_8_H_7_O_3_)_2_(C_10_H_8_N_2_)]·H_2_O
*M* *_r_* = 540.01
Monoclinic, 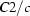

*a* = 19.888 (4) Å
*b* = 10.887 (2) Å
*c* = 11.612 (2) Å
β = 103.62 (3)°
*V* = 2443.5 (8) Å^3^
*Z* = 4
Mo *K*α radiation
μ = 0.94 mm^−1^
*T* = 293 K
0.1 × 0.1 × 0.1 mm

#### Data collection

Rigaku R-AXIS RAPID diffractometer
Absorption correction: multi-scan (*ABSCOR*; Higashi, 1995 ▶) *T*_min_ = 0.710, *T*_max_ = 0.780
12080 measured reflections
2796 independent reflections
2391 reflections with *I* > 2σ(*I*)
*R*_int_ = 0.054

#### Refinement

*R*[*F* ^2^ > 2σ(*F* ^2^)] = 0.041
*wR*(*F* ^2^) = 0.113
*S* = 1.05
2796 reflections
165 parameters
H-atom parameters constrained
Δρ_max_ = 0.35 e Å^−3^
Δρ_min_ = −0.39 e Å^−3^

### 

Data collection: *RAPID-AUTO* (Rigaku, 1998 ▶); cell refinement: *RAPID-AUTO*; data reduction: *CrystalStructure* (Rigaku/MSC, 2004 ▶); program(s) used to solve structure: *SHELXS97* (Sheldrick, 2008 ▶); program(s) used to refine structure: *SHELXL97* (Sheldrick, 2008 ▶); molecular graphics: *ORTEPII* (Johnson, 1976) ▶; software used to prepare material for publication: *SHELXL97*.

## Supplementary Material

Crystal structure: contains datablocks global, I. DOI: 10.1107/S1600536811005563/rn2078sup1.cif

Structure factors: contains datablocks I. DOI: 10.1107/S1600536811005563/rn2078Isup2.hkl

Additional supplementary materials:  crystallographic information; 3D view; checkCIF report

## Figures and Tables

**Table 1:** Hydrogen-bond geometry (Å, °)

*D*—H⋯*A*	*D*—H	H⋯*A*	*D*⋯*A*	*D*—H⋯*A*
O4—H41⋯O1	0.88	2.24	3.023 (3)	147
C12—H12*A*⋯O4^ii^	0.93	2.41	3.339 (3)	178
C11—H11*A*⋯O3^ii^	0.93	2.57	3.483 (3)	166
C10—H10*A*⋯O2^iii^	0.93	2.66	3.342 (3)	131

Symmetry codes: (ii) 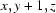; (iii) 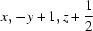.

**Fig. 1.**
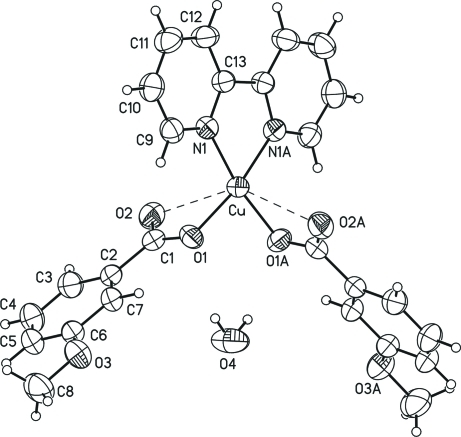
The molecular structure of the title compound,with atom labels and 45% probability displacement ellipsoids for non-H atoms. Symmetry code for the symbol 'A': -x, y + 1, 0.5 - z.

**Fig. 2.**
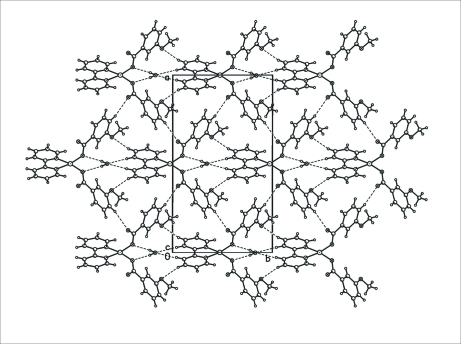
The two-dimensional supramolecular layers of the title compound parallel to (100) showing O–H···O, C–H···O hydrogen bonds.

## supplementary crystallographic information

### Comment

In the past decade, a variety of supramolecular architectures based on hydrogen bonds, π···π interactions have been achieved by using transition metal centers and organic ligands (Aakeroy *et al.*, 1993), they have potential application in catalysis, gas storage, and in molecular–based magnetic materials (Liu *et al.*, 2007, Shibasaki *et al.*, 2002). Herein, we are interested in self-assemblies of Cu^2+^ ions and bipy with 3–methoxybenzoic acid, which led to the preparation of [Cu(bipy)_2_(C_8_H_8_O_3_)_2_].H_2_O.

The title compound, [Cu(bipy)_2_(C_8_H_8_O_3_)_2_].H_2_O, is comprised of a Cu^II^ ion, two 3–methoxybenzoate ligands, a 2,2'–bipyridine(bipy) ligand and one lattice H_2_O molecule. As illustrated in Fig.1, the Cu ion and water O atom lie on a two fold axis. The Cu^II^ ion has a six–coordinate distorted octahedral geometry with two N atoms from the bipy ligand [Cu–N = 1.9996 (16) Å] and four O atoms from two 3–methoxybenzoate ligands [Cu–O = 1.9551 (15) and 2.6016 (16) Å]. Owing to geometric constraints and the Jahn–Teller effect, the Cu–O bonds in the axial direction are longer than in the equatorial plane. Two O atoms and two N atoms occupy the equatorial plane position with the r.m.s. deviation from the ideal plane of 0.214 Å, while two O atoms lie in the apical positions with an axis angle of 140.53 (5)° showing a large deviation from the normal 180°,which is also seen in similar carboxylate complexes (Liu *et al.*, 2010; Su *et al.*, 2005). For 3–methoxybenzoate anions, the plane of benzene ring and carboxylate group are nearly co–planar where the dihedral angle between the benzene ring and carboxylate plane is 5.2 (3)°. The water molecules are not coordinated to Cu and the distance between copper and water oxygen atoms is 4.019 (2) Å.

The molecules are linked *via* hydrogen bonds (O4–H41···O1, C12–H12A···O4, C11–H11A···O3) into one-dimensional supramolecular chains extending along the [100] direction, which are linked by hydrogen bonds (C5–H5A···O2) into two dimensional layers parallel to (100) (Fig. 2). The layers are arranged alternately in an ···ABAB···sequence and further assembled into there–dimensional network by hydrogen bonds (C10–H10A···O2).

### Experimental

CuCl_2_.2H_2_O (0.1705 g, 1.000 mmol) was successively added to 20 ml C_2_H_5_OH–H_2_O(1:1, *v*/*v*), 3–methoxybenzoate (0.1520 g, 1.000 mmol) and bipy (0.1569 g, 1.004 mmol) were subsequently added, then 1.4 ml (1 *M*) NaOH was added dropwise and stirred continuously for 1 h to give a blue suspension. After filtration, the blue filtrate (pH = 5.80) was allowed to stand at room temperature for several weeks to give blue block–shaped crystals

### Refinement

H atoms bonded to C atoms were placed in geometrically calculated positions and were refined using a riding model, with *U*_iso_(H) = 1.2 *U*_eq_(C). H atoms attached to O atoms were found in a difference Fourier synthesis and were refined using a riding model, with the O—H distances fixed as initially found and with *U*_iso_(H) values set at 1.2 *U*eq(O).

### Crystal data

**Table:**

[Cu(C_8_H_7_O_3_)_2_(C_10_H_8_N_2_)]·H_2_O	*F*(000) = 1116
*M**_r_* = 540.01	*D*_x_ = 1.468 Mg m^−^^3^
Monoclinic, *C*2/*c*	Mo *K*α radiation, λ = 0.71073 Å
Hall symbol: -C 2yc	Cell parameters from 12080 reflections
*a* = 19.888 (4) Å	θ = 3.6–27.5°
*b* = 10.887 (2) Å	µ = 0.94 mm^−^^1^
*c* = 11.612 (2) Å	*T* = 293 K
β = 103.62 (3)°	Block, blue
*V* = 2443.5 (8) Å^3^	0.1 × 0.1 × 0.1 mm
*Z* = 4	

### Data collection

**Table:**

Rigaku R-AXIS RAPID diffractometer	2796 independent reflections
Radiation source: fine-focus sealed tube	2391 reflections with *I* > 2σ(*I*)
graphite	*R*_int_ = 0.054
Detector resolution: 0 pixels mm^-1^	θ_max_ = 27.5°, θ_min_ = 3.6°
ω scans	*h* = −25→25
Absorption correction: multi-scan (*ABSCOR*; Higashi, 1995)	*k* = −14→14
*T*_min_ = 0.710, *T*_max_ = 0.78	*l* = −15→14
12080 measured reflections	

### Refinement

**Table:**

Refinement on *F*^2^	Primary atom site location: structure-invariant direct methods
Least-squares matrix: full	Secondary atom site location: difference Fourier map
*R*[*F*^2^ > 2σ(*F*^2^)] = 0.041	Hydrogen site location: inferred from neighbouring sites
*wR*(*F*^2^) = 0.113	H-atom parameters constrained
*S* = 1.05	*w* = 1/[σ^2^(*F*_o_^2^) + (0.0638*P*)^2^ + 0.7842*P*] where *P* = (*F*_o_^2^ + 2*F*_c_^2^)/3
2796 reflections	(Δ/σ)_max_ = 0.001
165 parameters	Δρ_max_ = 0.35 e Å^−^^3^
0 restraints	Δρ_min_ = −0.39 e Å^−^^3^

### Special details

**Table:**

Geometry. All e.s.d.'s (except the e.s.d. in the dihedral angle between two l.s. planes) are estimated using the full covariance matrix. The cell e.s.d.'s are taken into account individually in the estimation of e.s.d.'s in distances, angles and torsion angles; correlations between e.s.d.'s in cell parameters are only used when they are defined by crystal symmetry. An approximate (isotropic) treatment of cell e.s.d.'s is used for estimating e.s.d.'s involving l.s. planes.
Refinement. Refinement of *F*^2^ against ALL reflections. The weighted *R*-factor *wR* and goodness of fit *S* are based on *F*^2^, conventional *R*-factors *R* are based on *F*, with *F* set to zero for negative *F*^2^. The threshold expression of *F*^2^ > σ(*F*^2^) is used only for calculating *R*-factors(gt) *etc*. and is not relevant to the choice of reflections for refinement. *R*-factors based on *F*^2^ are statistically about twice as large as those based on *F*, and *R*- factors based on ALL data will be even larger.

### Fractional atomic coordinates and isotropic or equivalent isotropic displacement parameters (Å^2^)

**Table:**

	*x*	*y*	*z*	*U*_iso_*/*U*_eq_	
Cu	0.0000	0.52776 (3)	0.7500	0.04304 (15)	
O1	−0.04555 (7)	0.40392 (14)	0.82712 (14)	0.0539 (4)	
O2	−0.12489 (8)	0.44707 (15)	0.66421 (15)	0.0598 (4)	
O3	−0.15000 (9)	0.11263 (17)	1.05759 (15)	0.0669 (5)	
N1	−0.03034 (8)	0.66715 (15)	0.83787 (14)	0.0426 (4)	
C1	−0.10582 (10)	0.39069 (18)	0.75900 (19)	0.0470 (5)	
C2	−0.15286 (10)	0.30133 (18)	0.79947 (19)	0.0475 (5)	
C3	−0.21724 (12)	0.2726 (2)	0.7265 (2)	0.0611 (6)	
H3A	−0.2317	0.3089	0.6523	0.073*	
C4	−0.25923 (13)	0.1895 (3)	0.7658 (3)	0.0724 (7)	
H4A	−0.3021	0.1702	0.7170	0.087*	
C5	−0.23949 (12)	0.1344 (2)	0.8754 (3)	0.0646 (6)	
H5A	−0.2688	0.0790	0.9003	0.078*	
C6	−0.17571 (11)	0.16247 (19)	0.9479 (2)	0.0534 (5)	
C7	−0.13269 (10)	0.24562 (19)	0.9099 (2)	0.0498 (5)	
H7A	−0.0898	0.2643	0.9590	0.060*	
C8	−0.19197 (18)	0.0270 (3)	1.1014 (3)	0.0818 (9)	
H8A	−0.1674	−0.0022	1.1777	0.123*	
H8B	−0.2028	−0.0410	1.0476	0.123*	
H8C	−0.2340	0.0664	1.1085	0.123*	
C9	−0.06227 (11)	0.6578 (2)	0.92759 (18)	0.0492 (5)	
H9A	−0.0705	0.5802	0.9549	0.059*	
C10	−0.08304 (12)	0.7594 (2)	0.97979 (19)	0.0557 (5)	
H10A	−0.1055	0.7507	1.0411	0.067*	
C11	−0.07033 (13)	0.8737 (2)	0.9407 (2)	0.0599 (6)	
H11A	−0.0838	0.9434	0.9757	0.072*	
C12	−0.03730 (12)	0.8849 (2)	0.8490 (2)	0.0552 (5)	
H12A	−0.0282	0.9619	0.8215	0.066*	
C13	−0.01811 (10)	0.77943 (18)	0.79901 (17)	0.0426 (4)	
O4	0.0000	0.1586 (2)	0.7500	0.0842 (8)	
H41	−0.0138	0.2109	0.7975	0.126*	

### Atomic displacement parameters (Å^2^)

**Table:**

	*U*^11^	*U*^22^	*U*^33^	*U*^12^	*U*^13^	*U*^23^
Cu	0.0395 (2)	0.0333 (2)	0.0560 (2)	0.000	0.01071 (15)	0.000
O1	0.0423 (8)	0.0427 (8)	0.0740 (10)	−0.0074 (6)	0.0083 (7)	0.0071 (7)
O2	0.0532 (9)	0.0576 (9)	0.0692 (10)	0.0083 (7)	0.0159 (8)	0.0082 (8)
O3	0.0646 (10)	0.0641 (11)	0.0774 (11)	−0.0110 (8)	0.0277 (9)	0.0081 (8)
N1	0.0407 (9)	0.0405 (9)	0.0468 (9)	−0.0013 (7)	0.0108 (7)	0.0020 (6)
C1	0.0435 (11)	0.0356 (10)	0.0642 (12)	0.0055 (8)	0.0174 (9)	−0.0021 (9)
C2	0.0367 (10)	0.0361 (10)	0.0696 (13)	0.0015 (8)	0.0125 (9)	−0.0064 (9)
C3	0.0457 (12)	0.0546 (13)	0.0777 (15)	−0.0014 (10)	0.0040 (11)	−0.0038 (11)
C4	0.0400 (12)	0.0667 (16)	0.103 (2)	−0.0126 (11)	0.0024 (12)	−0.0111 (15)
C5	0.0459 (13)	0.0512 (13)	0.1009 (19)	−0.0111 (10)	0.0258 (12)	−0.0081 (12)
C6	0.0478 (12)	0.0425 (11)	0.0754 (14)	−0.0041 (9)	0.0254 (10)	−0.0069 (10)
C7	0.0383 (10)	0.0447 (11)	0.0671 (13)	−0.0052 (8)	0.0135 (9)	−0.0048 (9)
C8	0.093 (2)	0.0693 (19)	0.098 (2)	−0.0154 (15)	0.0526 (19)	0.0040 (14)
C9	0.0461 (11)	0.0513 (12)	0.0508 (11)	−0.0028 (9)	0.0127 (9)	0.0042 (9)
C10	0.0557 (13)	0.0657 (14)	0.0502 (11)	0.0017 (11)	0.0211 (10)	−0.0018 (10)
C11	0.0696 (15)	0.0536 (13)	0.0614 (13)	0.0079 (11)	0.0255 (11)	−0.0082 (10)
C12	0.0679 (14)	0.0386 (11)	0.0629 (13)	0.0042 (10)	0.0232 (11)	−0.0006 (9)
C13	0.0430 (10)	0.0384 (10)	0.0462 (10)	0.0013 (8)	0.0100 (8)	−0.0011 (7)
O4	0.124 (3)	0.0464 (14)	0.0887 (18)	0.000	0.0370 (17)	0.000

### Geometric parameters (Å, °)

**Table:**

Cu—O1^i^	1.9551 (15)	C5—C6	1.381 (3)
Cu—O1	1.9551 (15)	C5—H5A	0.9300
Cu—N1^i^	1.9996 (16)	C6—C7	1.387 (3)
Cu—N1	1.9996 (16)	C7—H7A	0.9300
O1—C1	1.279 (3)	C8—H8A	0.9600
O2—C1	1.239 (3)	C8—H8B	0.9600
O3—C6	1.368 (3)	C8—H8C	0.9600
O3—C8	1.423 (3)	C9—C10	1.371 (3)
N1—C13	1.345 (2)	C9—H9A	0.9300
N1—C9	1.346 (3)	C10—C11	1.369 (3)
C1—C2	1.500 (3)	C10—H10A	0.9300
C2—C7	1.390 (3)	C11—C12	1.382 (3)
C2—C3	1.395 (3)	C11—H11A	0.9300
C3—C4	1.380 (4)	C12—C13	1.380 (3)
C3—H3A	0.9300	C12—H12A	0.9300
C4—C5	1.378 (4)	C13—C13^i^	1.483 (4)
C4—H4A	0.9300	O4—H41	0.8800
			
O1^i^—Cu—O1	92.80 (10)	O3—C6—C7	115.6 (2)
O1^i^—Cu—N1^i^	93.53 (7)	C5—C6—C7	119.8 (2)
O1—Cu—N1^i^	170.12 (6)	C6—C7—C2	120.8 (2)
O1^i^—Cu—N1	170.12 (6)	C6—C7—H7A	119.6
O1—Cu—N1	93.53 (7)	C2—C7—H7A	119.6
N1^i^—Cu—N1	81.25 (9)	O3—C8—H8A	109.5
C1—O1—Cu	105.20 (13)	O3—C8—H8B	109.5
C6—O3—C8	118.1 (2)	H8A—C8—H8B	109.5
C13—N1—C9	118.97 (17)	O3—C8—H8C	109.5
C13—N1—Cu	114.74 (12)	H8A—C8—H8C	109.5
C9—N1—Cu	126.27 (14)	H8B—C8—H8C	109.5
O2—C1—O1	122.66 (19)	N1—C9—C10	121.83 (19)
O2—C1—C2	121.1 (2)	N1—C9—H9A	119.1
O1—C1—C2	116.23 (18)	C10—C9—H9A	119.1
C7—C2—C3	119.2 (2)	C11—C10—C9	119.26 (19)
C7—C2—C1	120.43 (19)	C11—C10—H10A	120.4
C3—C2—C1	120.4 (2)	C9—C10—H10A	120.4
C4—C3—C2	119.1 (2)	C10—C11—C12	119.6 (2)
C4—C3—H3A	120.4	C10—C11—H11A	120.2
C2—C3—H3A	120.4	C12—C11—H11A	120.2
C5—C4—C3	121.9 (2)	C13—C12—C11	118.6 (2)
C5—C4—H4A	119.1	C13—C12—H12A	120.7
C3—C4—H4A	119.1	C11—C12—H12A	120.7
C4—C5—C6	119.2 (2)	N1—C13—C12	121.68 (17)
C4—C5—H5A	120.4	N1—C13—C13^i^	114.60 (10)
C6—C5—H5A	120.4	C12—C13—C13^i^	123.71 (12)
O3—C6—C5	124.6 (2)		

Symmetry codes: (i) −*x*, *y*, −*z*+3/2.

### Hydrogen-bond geometry (Å, °)

**Table:**

*D*—H···*A*	*D*—H	H···*A*	*D*···*A*	*D*—H···*A*
O4—H41···O1	0.88	2.24	3.023 (3)	147
C12—H12A···O4^ii^	0.93	2.41	3.339 (3)	178
C11—H11A···O3^ii^	0.93	2.57	3.483 (3)	166
C10—H10A···O2^iii^	0.93	2.66	3.342 (3)	131

Symmetry codes: (ii) *x*, *y*+1, *z*; (iii) *x*, −*y*+1, *z*+1/2.
